# Associations between serum uric acid levels and hypotension after general anesthesia induction in hypertensive patients

**DOI:** 10.1590/1414-431X2025e14729

**Published:** 2025-10-06

**Authors:** Yahong Zhang, Jialu Wan, Guikuan Nie

**Affiliations:** 1 Department of Anesthesiology, Chengdu Seventh People's Hospital (Affiliated Cancer Hospital of Chengdu Medical College), Chengdu, China; 2 Geriatric Diseases Institute of Chengdu/Cancer Prevention and Treatment Institute of Chengdu, Department of Anesthesiology, Chengdu Fifth People's Hospital (The Second Clinical Medical College, Affiliated Fifth People's Hospital of Chengdu University of Traditional Chinese Medicine), Chengdu, China; 3 Department of Colorectal and Anal Surgery, Chengdu Seventh People's Hospital (Affiliated Cancer Hospital of Chengdu Medical College), Chengdu, China

**Keywords:** Hypertension, Blood uric acid, PIH, Anesthesia

## Abstract

Postinduction hypotension (PIH), a common complication of general anesthesia, occurs more frequently in hypertensive patients. This hypotensive state may induce hypoxia in vital organs, potentially progressing to organ dysfunction or even death. In this prospective cohort study, the primary outcome was incidence of PIH. The preanesthesia baseline parameters included demographic characteristics (age, sex, and body mass index [BMI]) and laboratory biomarkers (serum uric acid, hemoglobin, and lipoprotein levels). Noninvasive blood pressure was systematically monitored at five time points: preinduction (T_0_), postinduction (T_1_), immediately postintubation (T_2_), 5 min postintubation (T_3_), and 10 min postintubation (T_4_). This study involved 271 hypertensive patients with a median age (interquartile range) of 56.0 (17.0) years. The cohort comprised 134 males (49.4%) with a mean BMI of 23.8±2.94 kg/m^2^. PIH occurred in 165 patients (60.8%) following general anesthesia. Univariate logistic regression revealed potential associations between PIH and serum uric acid levels, advanced age, elevated baseline systolic blood pressure, grade 3 hypertension, fasting duration, and ASA class III status. Multivariate logistic regression suggested that serum uric acid may exert a protective effect, whereas grade 3 hypertension, age, and baseline systolic blood pressure emerged as risk modulators. Notably, a composite model incorporating age, baseline systolic blood pressure, hypertension severity, and serum uric acid level demonstrated enhanced predictive capacity (AUC=0.863 *vs* 0.712 for serum uric acid level alone, both P<0.01). Serum uric acid level demonstrated a moderate inverse correlation with PIH, whereas grade 3 hypertension, age, and baseline systolic blood pressure emerged as potential risk factors for PIH occurrence.

## Introduction

Postinduction hypotension (PIH), defined as a systolic blood pressure <90 mmHg or ≥30% decrease in mean arterial pressure within 20 min following anesthesia induction (1), is a prevalent complication of general anesthesia. Its incidence ranges from 45-55%, with variations attributable to patient demographics, surgical complexity, and anesthetic regimens (2,3). High-risk subgroups, including cardiovascular-compromised elderly patients and prolonged preoperative fasting populations, have elevated incidence rates (4,5). PIH induces cerebral hypoxia, myocardial ischemia, cardiovascular dysfunction, and pulmonary compromise, and prolongs the duration of postoperative convalescence, extends the hospitalization duration, and increases healthcare expenditures (6,7). Hypertensive cohorts demonstrate a two-fold greater PIH susceptibility due to endothelial dysfunction and target-organ baroreceptor impairment, which predisposes patients to hemodynamic instability (8,9). Consequently, implementing preoperative risk stratification through multidimensional assessment, including demographic characteristics and laboratory biomarkers, enables evidence-based preventive protocols, which are critical for reducing intraoperative hypotensive episodes and optimizing postoperative recovery trajectories (10).

Uric acid, the end product of 2,6,8-trioxypurine metabolism, originates primarily from nucleic acid catabolism and undergoes renal-hepatic clearance (11,12). The pathophysiological relationship between uric acid concentrations and PIH remains incompletely characterized. Emerging clinical evidence suggests an inverse association between serum uric acid levels and general anesthesia-induced hypotensive events. Deepshikha et al. (13) demonstrated that early preeclamptic women with hypouricemia exhibit a greater incidence of spinal anesthesia-induced hypotension than do those with hyperuricemia. Similarly, Nidhi et al. (14) reported that, compared with normouricemic controls, normotensive parturients who underwent emergency cesarean delivery with elevated serum uric acid levels presented a lower incidence of spinal anesthesia-induced hypotension and required fewer vasopressor boluses. Notably, evidence regarding the prognostic value of serum uric acid level for general anesthesia-induced PIH in hypertensive individuals remains limited, constituting a critical knowledge gap.

Therefore, we conducted this multicenter trial to investigate the association between serum uric acid levels and the incidence of PIH.

## Material and Methods

### Study design and participants

This multicenter prospective cohort study was conducted across three tertiary hospitals (Chengdu Seventh People's Hospital (Affiliated Cancer Hospital of Chengdu Medical College), Chengdu Fifth People's Hospital, and Chengdu Pidu District People's Hospital) and received ethical approval from the institutional review boards/ethics committees of all participating centers. Prior to study initiation, the protocol was registered with the Chinese Clinical Trial Registry (ChiCTR2200058036). All participants provided written informed consent before enrolment.

The inclusion criteria were: 1) primary hypertension diagnosis; 2) regular antihypertensive medication for ≥3 months preoperatively; 3) age ≥18 years; 4) American Society of Anesthesiologists (ASA) physical status I-III; and 5) scheduled for general anesthesia.

The exclusion criteria were: 1) poorly controlled hypertension (preoperative baseline blood pressure (BP) ≥180/110 mmHg); 2) severe hypertensive complications (heart failure, cerebral hemorrhage, or stroke); 3) continued use of ACE inhibitors, ARBs, or diuretics on surgery day; 4) participation in other clinical trials within 3 months; 5) pregnancy; 6) substance abuse disorders; 7) active psychiatric conditions; 8) severe hepatic/renal dysfunction; and 9) severe cardiopulmonary comorbidities, significant valvulopathy, congenital heart disease, peripheral vascular disease, or morbid obesity.

Study withdrawal triggers included the following: 1) anesthetic drug hypersensitivity; 2) unanticipated difficult airway; and 3) major anesthesia protocol deviation.

### Participant assessment

During the preoperative evaluation, baseline demographic characteristics, including age, sex, body mass index (BMI), hypertension duration, hypertension stage, current antihypertensive medications, and surgical category, were collected. The following perioperative parameters were systematically recorded: serum uric acid concentration, baseline systolic blood pressure, fasting duration (hours), ASA physical status classification, preanesthesia intravenous fluid administration volume, intraoperative ephedrine cumulative dose, admission uric acid levels, low-density lipoprotein, high-density lipoprotein, total cholesterol, and hemoglobin levels, and other clinically relevant parameters.

### Sample size calculation

Previous studies reported a 41.2% incidence of PIH in hyperuricemic patients *versus* 61.2% in normouricemic controls (15). The standard sample size formula for comparative studies was n=2*pq* (*U*_α_+*U*_β_) / (*p*_1_-*p*_0_)^2^, where *p*_1_=0.412 (exposed group incidence), *p*_0_=0.612 (nonexposed incidence), *q*=1-*p*, α=0.05 (two-tailed), β=0.10, *U*α=1.96, and *U*_β_=1.282, and the calculated minimum sample size per group was 82 participants. Accounting for a 15% attrition rate, the total required enrollment was 189 patients.

### Study procedure

Twenty-four hours preoperatively, anesthesiology team members systematically reviewed the electronic health records of candidates fulfilling the inclusion criteria after completing mandatory preoperative assessments. These assessments comprised standardized laboratory panels: a comprehensive metabolic panel (hepatic/renal functions), a coagulation profile, and a complete blood count. Subsequently, structured preoperative anesthesia consultations were conducted in patient wards to verify medical histories and optimize perioperative management. Written informed consent was obtained from all study participants through standardized documentation procedures.

Following the application of standard perioperative monitoring (electrocardiogram [ECG], pulse oximetry [SpO_2_], and noninvasive blood pressure), a 20-gauge *iv* cannula was inserted in the operating suite. Initiation of compound sodium chloride infusion (5 mL/kg) commenced immediately, with complete administration achieved within 15 min preceding anesthetic induction.

Anesthesia induction was achieved via the use of midazolam (0.04 mg/kg), sufentanil (0.3 μg/kg), propofol (1.5 mg/kg), and rocuronium (0.9 mg/kg). Anesthesia maintenance between intubation and the surgical incision was achieved through continuous infusion of propofol (4 mg/kg/h) and remifentanil (0.1 μg/kg/min). A supplemental sufentanil bolus (0.1 μg/kg) was administered before incision. The depth of anesthesia was titrated to maintain bispectral index (BIS) values of 40-60 through continuous infusions of propofol (6-8 mg/kg/h) and remifentanil (0.1-0.2 μg/kg per min) supplemented with sevoflurane (2-3% end-tidal concentration).

Rocuronium was administered intermittently as needed until 30 min prior to surgical conclusion, with sevoflurane inhalation discontinued 15 min before procedure completion. Propofol and remifentanil infusions were terminated upon skin closure. Following confirmation of full consciousness and fulfilment of extubation criteria, the endotracheal tube was removed, accompanied by standard neuromuscular blockade reversal using neostigmine (0.04 mg/kg) and atropine (0.01 mg/kg). Patients were transferred to the post-anesthesia care unit (PACU), with ward transfer initiated when the Steward Recovery Scores exceeded 4 points. Hemodynamic management included intravenous ephedrine (6 mg) for hypotension (BP<90/60 mmHg), urapidil (20 mg) for hypertension (BP>180/110 mmHg), and repeatable atropine (0.25 mg) for bradycardia (heart rate<50 bpm). BP was systematically recorded at preinduction (T_0_), postinduction (T_1_), immediately postintubation (T_2_), and at 5-min (T_3_) and 10-min (T_4_) intervals postintubation.

### Diagnostic and grading criteria

#### Hypertension classification

Blood pressure was stratified into three grades: Grade 1 (systolic BP (SBP) 140-159 mmHg and/or diastolic BP (DBP) 90-99 mmHg), Grade 2 (SBP 160-179 mmHg and/or DBP 100-109 mmHg), and Grade 3 (SBP≥180 mmHg and/or DBP≥110 mmHg).

#### Uric acid level stratification

Following the 2015 American College of Rheumatology (ACR)/European League Against Rheumatism (EULAR) Gout Classification Criteria (16), the serum urate levels were categorized into five tiers: <240, 240-<360, 360-<480, 480-<600, and ≥600 μmol/L.

#### Postinduction hypotension quantification

The magnitude of blood pressure reduction after anesthesia induction (ΔMAP) was calculated as follows: ΔMAP = [(MAP_T0_ - MAP_T1_) / MAP_T0_] × 100% where MAP_T0_ denotes the baseline mean arterial pressure and MAP_T1_ indicates the postinduction value.

### Data analysis

Statistical analyses were performed using SPSS software (version 22.0; IBM Corp., USA). Normally distributed continuous variables are reported as means±SD and were compared using independent-samples *t*-tests. Non-normally distributed variables are reported as medians (interquartile ranges), and Mann-Whitney U tests were used for group comparisons.

Categorical variables, including sex, ASA classification, antihypertensive regimen, diabetes complications, and surgical type, were summarized as counts (percentages) and analyzed using χ^2^ tests.

Univariate logistic regression was used to identify potential predictors of the primary outcome, and variables with P<0.01 were subsequently entered into multivariate logistic regression models. Statistical significance was defined as a two-tailed P-value <0.05.

## Results

### Baseline and clinical characteristics of the participants

**Figure 1:**
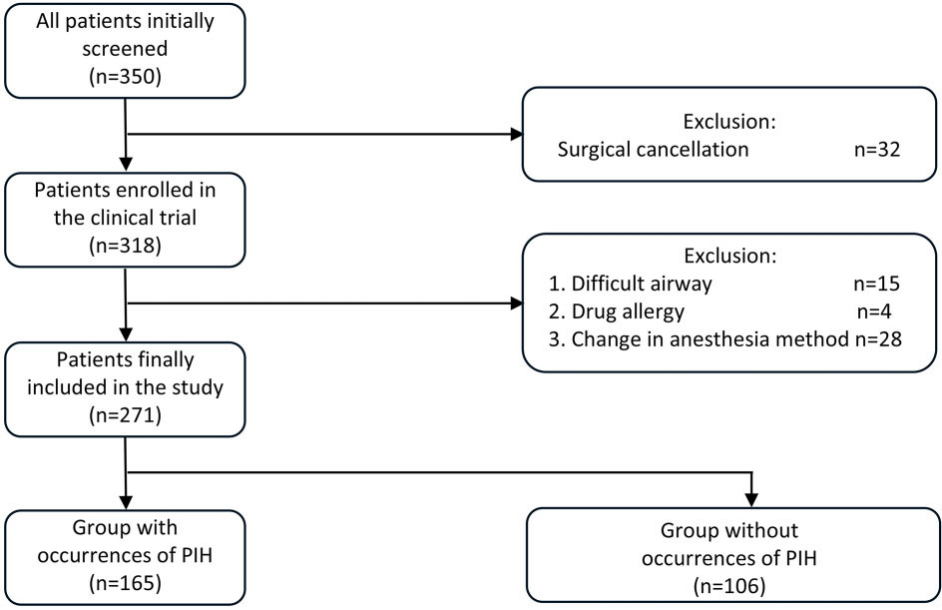
Flowchart of the study. PIH: postinduction hypotension.

Between January and December 2022, 350 hypertensive patients scheduled for elective surgery under general anesthesia were initially assessed. The exclusion of patients was due to 32 cases with cancelled procedures, 15 cases of difficult airway management, 4 drug hypersensitivity reactions, and 28 protocol deviations due to anesthesia scheme modifications. The final cohort included 271 patients stratified into PIH and non-PIH groups for analysis (Figure 1).

The median age with interquartile range was comparable between the groups at 56.0 (17.0) years, comprising 134 male patients (49.4%) with a mean BMI of 23.8±2.9 kg/m^2^. Antihypertensive regimens predominantly included calcium channel blockers (CCBs) in 145 patients (50.7%), β-blockers in 93 patients (32.5%), ACE inhibitors (ACEIs) in 38 patients (13.3%), and diuretics in 10 patients (3.5%). No intergroup differences emerged in sex distribution, BMI, hypertension duration, or medication regimens (all P>0.05). However, disparities were observed regarding age, ASA classification, baseline systolic pressure, serum uric acid levels, and ephedrine administration (P<0.05) (Table 1).

**Table 1:** Comparison of general characteristics between groups with and without postinduction hypotension (PIH) after anesthetic induction in hypertensive patients.

	PIH group (n=165)	Non-PIH group (n=106)	P
Age (years)	60.0 (18.0)	52.0 (15.0)	0.045
Gender (male)	79 (59.0%)	55 (41.0%)	0.801
BMI (kg/m^2^)	23.3±3.3	24.6±2.1	0.248
ASA, n (%)			0.044
I	37 (22.4%)	30 (28.3%)	
II	89 (53.9%)	65 (61.3%)	
III	39 (23.6%)	11 (10.4%)	
History of hypertension (years)	11.0 (6.0)	9.0 (7.0)	0.584
Anti-hypertensive regimens, n (%)			0.823
CCBs	89 (51.4%)	56 (49.6%)	
β-blockers	56 (32.4%)	37 (32.7%)	
ACEIs	23 (13.3%)	15 (13.3%)	
Diuretics	5 (2.9%)	5 (4.4%)	
Baseline SBP (mmHg)	163.2±6.2	135.7±7.8	0.039
Uric acid (μmol/L)	309.65±7.9	488.7±16.2	0.036
Type 2 diabetes, n (%)	65 (39.4%)	34 (32.1%)	0.738
Surgical type, n (%)			0.086
Orthopedics	12 (30.8%)	23 (69.2%)	
Urology	22 (50.0%)	22 (50.0%)	
General surgery	23 (56.1%)	17 (43.9%)	
Gastroenterology	6 (35.3%)	11 (64.7%)	
Hepatobiliary	21 (85.7%)	4 (14.3%)	
Neurosurgery	2 (13.3%)	13 (86.7%)	
Otolaryngology	6 (42.9%)	8 (57.1%)	
Amount of ephedrine (mg)	8.2±3.4	2.5±2.3	0.012

Data are reported as means and SD or number and percentage. BMI: body mass index; ASA: American Society of Anesthesiologists; CCB: calcium channel blockers; ACEI: angiotensin converting enzyme inhibitors; SBP: systolic blood pressure.

### Serum uric acid and the incidence of PIH

**Figure 2:**
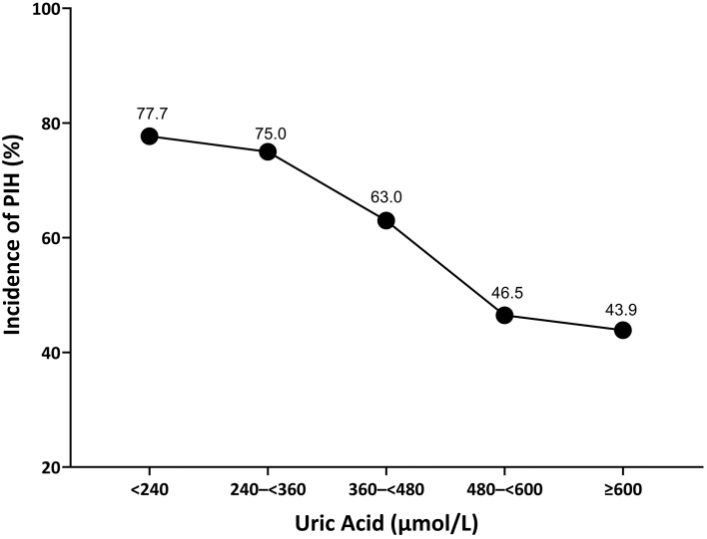
Relationship between serum uric acid and postinduction hypotension (PIH) in hypertensive patients.

Serum uric acid levels ranged from 108 μmol/L (minimum) to 754 μmol/L (maximum). We observed a moderate inverse relationship between serum uric acid concentrations and incidence of PIH. The PIH rates demonstrated dynamic patterns across quintile intervals: 77.7% (<240 μmol/L), 75.0% (240-<360 μmol/L), 63.0% (360-<480 μmol/L), 46.5% (480-<600 μmol/L), and 43.9% (≥600 μmol/L) (Figure 2).

### Serum uric acid levels and the degree of blood pressure drop after anesthetic induction

**Figure 3:**
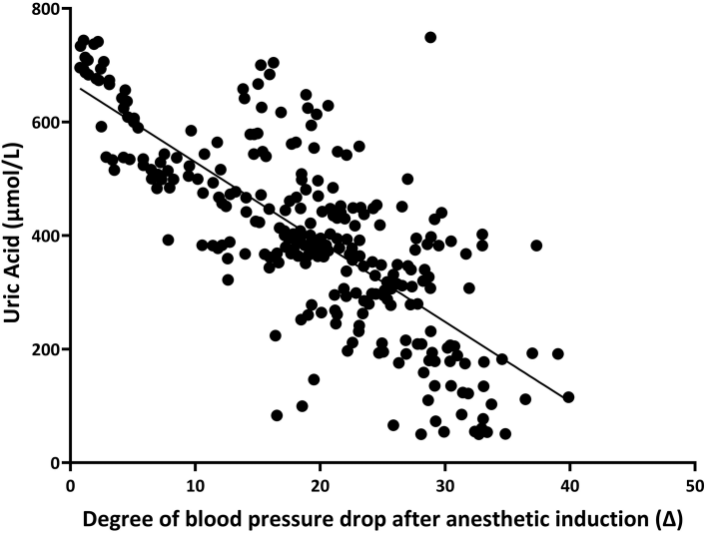
Linear regression between serum uric acid levels and the degree of blood pressure drop after anesthetic induction (Δ) in hypertensive patients.

Linear regression analysis was used to quantify the relationship between preoperative serum uric acid concentrations and the degree of decrease in blood pressure after anesthetic induction (Δ). The model demonstrated moderate explanatory power (R^2^=0.582), with scatterplot visualization confirming a moderate inverse correlation between elevated uric acid levels and diminished Δ (P<0.05) (Figure 3).

### Serum uric acid levels and ephedrine dosage

**Figure 4:**
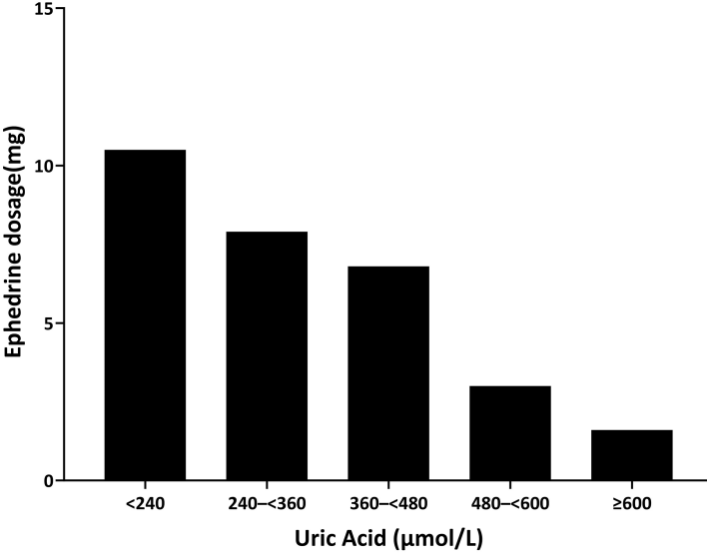
Relationship between serum uric acid levels and ephedrine dosage at recovery from general anesthesia in hypertensive patients.

**Figure 5:**
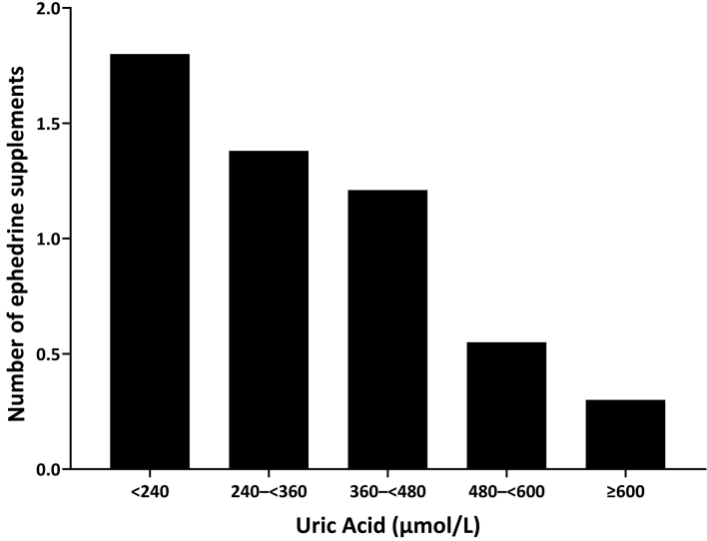
Relationship between serum uric acid levels and the number of ephedrine doses at recovery from general anesthesia in hypertensive patients.

Patients with serum uric acid levels <240 μmol/L had increased ephedrine requirements (mean dose: 10.5±3.3 mg; mean number of administrations: 1.8±0.4), whereas those with uric acid levels >600 μmol/L had reduced usage (dose: 1.6±2.3 mg; number of administrations: 0.3±0.6) (Figures 4 and 5).

### Univariate analysis of PIH

PIH occurred in 165 patients (60.9%). Univariate analysis identified six risk predictors: age (OR=1.037, 95%CI: 1.008-1.068), baseline systolic blood pressure (OR=1.089, 95%CI: 1.064-1.115), grade 3 hypertension (OR=3.071, 95%CI: 1.511-6.244), fasting duration (OR=1.179, 95%CI: 1.023-1.358), ASA grade III classification (OR=2.118, 95%CI: 1.153-3.891), and serum uric acid levels (OR=0.995, 95%CI: 0.993-0.997), with all associations meeting statistical significance (P<0.05) (Table 2).

**Table 2 t02:** Univariate analysis of risk factors for postinduction hypotension (PIH) after anesthetic induction in hypertensive patients.

Indicators	β	OR (95%CI)	P
Age (years)	0.037	1.037 (1.008-1.068)	0.013
Gender (male)	-0.434	0.648 (0.397-1.058)	0.083
BMI (kg/m^2^)	-0.092	0.912 (0.778-1.070)	0.259
Baseline systolic blood pressure (mmHg)	0.085	1.089 (1.064-1.115)	0.000
Duration of hypertension (years)	-0.002	0.998 (0.942-1.057)	0.945
Hypertension grading			
Grade 1		1	
Grade 2	-0.131	0.878 (0.504-1.528)	0.644
Grade 3	1.122	3.071 (1.511-6.244)	0.002
Fasting duration (hours)	-0.014	0.986 (0.880-1.105)	0.809
Duration of NPO (hours)	0.164	1.179 (1.023-1.358)	0.023
ASA			
I		1	
II	0.472	1.604 (0.887-2.9)	0.118
III	0.750	2.118 (1.153-3.891)	0.016
Preoperative intravenous fluid volume (mL)	-0.001	0.999 (0.998-1.000)	0.110
Uric acid (μmol/L)	-0.005	0.995 (0.993-0.997)	0.000
Low-density lipoprotein (mmol/L)	-0.249	0.780 (0.364-1.669)	0.522
High-density lipoprotein (mmol/L)	-0.418	0.659 (0.250-1.733)	0.398
Total cholesterol (mmol/L)	-0.045	0.956 (0.500-1.829)	0.892
Hemoglobin (g/L)	0.004	1.004 (0.986-1.023)	0.633

BMI: body mass index; ASA: American Society of Anesthesiologists; Duration of NPO: time a patient is not allowed to eat or drink.

### Multivariate analysis of PIH

Following preliminary screening, variables demonstrating statistical significance in univariate logistic regression were incorporated into multivariable analysis. As summarized in Tables 1-3, age (OR=1.045, 95%CI: 1.006-1.085), baseline systolic blood pressure (OR=1.090, 95%CI: 1.063-1.118), and grade 3 hypertension (OR=2.668, 95%CI: 1.113-6.398) emerged as risk factors for PIH (P<0.05). Notably, elevated uric acid levels exhibited a moderate inverse association with PIH in hypertensive patients (OR=0.995, 95%CI: 0.993-0.997, P<0.05) Table 3.

**Table 3:** Multivariate analysis of factors influencing postinduction hypotension (PIH) after anesthetic induction in hypertensive patients.

Indicators	β	OR (95%CI)	P
Age (years)	0.044	1.045 (1.006-1.085)	0.023
Baseline systolic blood pressure (mmHg)	0.086	1.090 (1.063-1.118)	0.000
Hypertension Grading			
Grade 1		1	
Grade 2	-0.170	0.844 (0.414-1.722)	0.641
Grade 3	0.981	2.668 (1.113-6.398)	0.028
Duration of NPO (hours)	0.143	1.154 (0.953-1.397)	0.142
ASA classification			
Class I		1	
Class II	0.374	1.454 (0.684-3.09)	0.330
Class III	0.764	2.147 (0.969-4.758)	0.060
Uric acid (μmol/L)	-0.005	0.995 (0.993-0.997)	0.000

ASA: American Society of Anesthesiologists; Duration of NPO: time a patient is not allowed to eat or drink.

### Receiver operating characteristic curves

**Figure 6:**
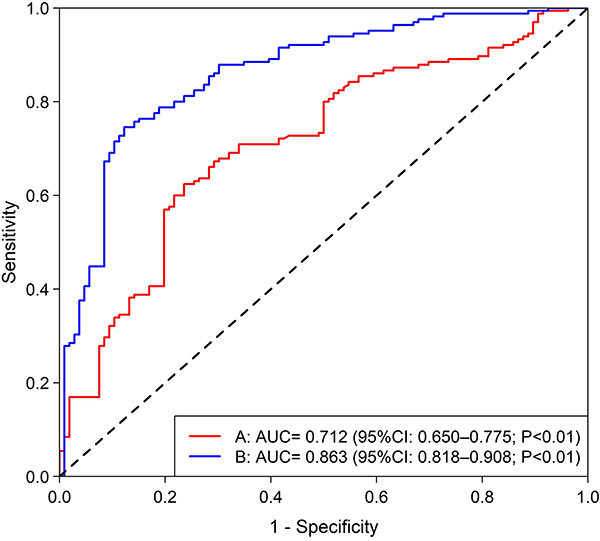
Red line (**A**): ROC curve of serum uric acid alone for predicting postinduction hypotension (PIH) (AUC= 0.712 (95%CI: 0.650-0.775; P<0.01). Blue line (**B**): ROC curve of serum uric acid combined with other variables (age, baseline systolic blood pressure, hypertension grade) for predicting postinduction hypotension (PIH) (AUC=0.863, 95%CI: 0.818-0.908; P<0.01).

Receiver operating characteristic (ROC) curves were constructed to evaluate the diagnostic performance of serum uric acid level alone and in combination with age, baseline systolic blood pressure, and hypertension grade for predicting PIH (Figure 6). Serum uric acid level alone had an area under the curve (AUC) of 0.712 (95%CI: 0.650-0.775; P<0.01). The combined model incorporating age, baseline systolic blood pressure, hypertension grade, and serum uric acid level demonstrated enhanced discriminative ability (AUC=0.863, 95%CI: 0.818-0.908; P<0.01).

## Discussion

In this study, a linear regression model was used to assess the correlation between uric acid levels and the reduction in blood pressure (Δ) after anesthesia induction. The model yielded an R^2^ value of 0.582, suggesting a moderate negative correlation between uric acid levels and the degree of blood pressure reduction (Δ). This finding indicated that uric acid level was inversely associated with PIH in hypertensive patients, where higher uric acid levels corresponded to less pronounced blood pressure reduction following anesthesia induction. A potential mechanistic explanation involves competitive inhibition of nitric oxide (NO) synthesis by uric acid. NO activates guanylate cyclase, thereby promoting intracellular cyclic guanosine monophosphate (cGMP) production. Elevated cGMP reduces the intracellular free calcium ion concentration, resulting in vascular smooth muscle relaxation, vasodilation, and subsequent lowering of blood pressure (17-20).

Simultaneously, elevated uric acid levels may directly impair endothelial cell function, suppress NO synthase activity, and thereby reduce NO production (21,22). Furthermore, excessive uric acid can promote oxidative stress or enzymatic degradation of NO, further diminishing its bioavailability (23). Additionally, uric acid has been shown to activate the renin-angiotensin system (RAS) by upregulating the expression of angiotensin I and II genes, thereby inducing local RAS stimulation (24,25). Moreover, uric acid exerts direct pro-sclerotic effects on renal afferent arteriolar smooth muscle and endothelial cells, contributing to vascular stiffening. It also disrupts renal sodium handling, increasing sodium retention and potentiating sodium-dependent hypertension (26-28).

This study revealed that 165 (60.9%) hypertensive patients developed hypotension following general anesthesia induction. This incidence is lower than the rates reported in previous studies on PIH (29,30). This discrepancy may be attributable to differences in study populations and protocols: our cohort specifically included hypertensive patients undergoing general anesthesia, whereas prior investigations primarily involved normotensive individuals receiving spinal anesthesia, with additional constraints on age and ASA physical status classification (29,30).

Severe hypertension is an independent risk factor for PIH, which may arise through interconnected mechanisms. Specifically, hypertensive patients often develop left ventricular hypertrophy and impaired diastolic function, compromising cardiac compensatory capacity during hemodynamic stress. Furthermore, anesthetic drugs administered during induction promote arterial vasodilation, thereby reducing systemic vascular resistance (31,32). Additionally, chronic hypertension is associated with arteriosclerosis and diminished vascular elasticity, both of which impair systemic vascular resistance regulation and increase susceptibility to hemodynamic instability (33).

This study revealed that advanced age was associated with an increased incidence of PIH, establishing age as an independent risk factor for this complication. This association may be attributed to age-related physiological changes, including reduced vascular wall elasticity and impaired contractile function (34,35). These alterations heighten vascular sensitivity to pharmacological agents, increasing susceptibility to vasodilation and subsequent hypotension. Notably, research by Gunin et al. (36) demonstrated that ageing correlates with reduced dermal vascular fibroblast density and Piezo1 expression, which compromises postanesthesia blood pressure regulation in older adults. Moreover, autonomic nervous system dysfunction - characterized by diminished sympathetic and parasympathetic regulation - progresses with age, impairing compensatory responses to hemodynamic challenges and exacerbating the risk of hypotension. Additionally, age-related declines in hepatic and renal function may delay drug metabolism and clearance; prolonged drug retention can increase plasma concentrations, potentiating hypotensive effects. Therefore, vigilant hemodynamic monitoring and proactive interventions are critical in older patients during anesthesia recovery to mitigate postinduction hypotension.

Elevated baseline systolic blood pressure represents a potential independent risk factor for PIH. This association may stem from its correlation with vascular dysfunction, particularly arterial stiffness - a vascular pathology characterized by loss of arterial wall elasticity and impaired contractile capacity (37,38). Notably, elevated systolic pressure frequently reflects underlying arterial stiffening, which collectively impairs cardiovascular autoregulation, heightens pharmacological sensitivity, and amplifies hemodynamic instability during anesthesia induction (39,40).

This study had several limitations. First, arterial stiffness parameters were not assessed because of equipment availability constraints and protocol design. Given the potential role of vascular compliance in hemodynamic instability during anesthesia induction, this omission may partially limit the mechanistic interpretation of the observed association between elevated baseline systolic blood pressure and PIH. Second, the lack of standardized diagnostic criteria for PIH complicates cross-study comparisons, potentially affecting external validity. Third, blood pressure was recorded at 5-min intervals postinduction, which may have led to missed transient hypotensive episodes; future studies should integrate continuous hemodynamic monitoring to improve accuracy. Finally, while we analyzed key variables (e.g., age, baseline blood pressure, uric acid levels), unmeasured confounders such as surgical complexity, preoperative medications, and fluid management strategies were not comprehensively addressed. Expanded cohorts with rigorous control of these factors are needed to clarify the role of uric acid in PIH pathogenesis.

## Conclusion

Serum uric acid levels have a moderate inverse association with PIH occurrence in hypertensive individuals. Notably, stage 3 hypertension, advanced age, and elevated baseline systolic blood pressure were independently associated with increased PIH risk.
